# Unique Features of the Gut Microbiome Characterized in Animal Models of Angelman Syndrome

**DOI:** 10.1128/msystems.00608-22

**Published:** 2023-01-04

**Authors:** Ulrika Beitnere, Brayan Vilanova-Cuevas, Sarah G. Christian, Clint Taylor, Elizabeth L. Berg, Nycole A. Copping, Scott V. Dindot, Jill L. Silverman, Mélanie G. Gareau, David J. Segal

**Affiliations:** a Genome Center, University of California Davis, Davis, California, USA; b Department of Anatomy, Physiology and Cell Biology, School of Veterinary Medicine, University of California Davis, Davis, California, USA; c Department of Veterinary Pathobiology, Texas A&M University, College Station, Texas, USA; d Department of Psychiatry and Behavioral Sciences, University of California Davis, Davis, California, USA; e Department of Biochemistry and Molecular Medicine, University of California Davis, Davis, California, USA; Duke University School of Medicine

**Keywords:** Angelman syndrome, neurodevelopmental disorders, 16S rRNA gene sequencing, gut-brain axis, microbiota, animal models, bacterial metabolites, gut microbiome

## Abstract

A large subset of patients with Angelman syndrome (AS) suffer from concurrent gastrointestinal (GI) issues, including constipation, poor feeding, and reflux. AS is caused by the loss of ubiquitin ligase E3A (*UBE3A*) gene expression in the brain. Clinical features of AS, which include developmental delays, intellectual disability, microcephaly, and seizures, are primarily due to the deficient expression or function of the maternally inherited *UBE3A* allele. The association between neurodevelopmental delay and GI disorders is part of the increasing evidence suggesting a link between the brain and the gut microbiome via the microbiota-gut-brain axis. To investigate the associations between colonization of the gut microbiota in AS, we characterized the fecal microbiome in three animal models of AS involving maternal deletions of *Ube3A*, including mouse, rat, and pig, using 16S rRNA amplicon sequencing. Overall, we identified changes in bacterial abundance across all three animal models of AS. Specific bacterial groups were significantly increased across all animal models, including *Lachnospiraceae* Incertae sedis, *Desulfovibrios* sp., and *Odoribacter*, which have been correlated with neuropsychiatric disorders. Taken together, these findings suggest that specific changes to the local environment in the gut are driven by a *Ube3a* maternal deletion, unaffected by varying housing conditions, and are prominent and detectable across multiple small and large animal model species. These findings begin to uncover the underlying mechanistic causes of GI disorders in AS patients and provide future therapeutic options for AS patients.

**IMPORTANCE** Angelman syndrome (AS)-associated gastrointestinal (GI) symptoms significantly impact quality of life in patients. In AS models in mouse, rat, and pig, AS animals showed impaired colonization of the gut microbiota compared to wild-type (healthy) control animals. Common changes in AS microbiomes across all three animal models may play a causal effect for GI symptoms and may help to identify ways to treat these comorbidities in patients in the future.

## INTRODUCTION

Angelman syndrome (AS) is a rare (1 in 15,000 births) genetic neurodevelopmental syndrome caused by the loss of maternally inherited ubiquitin ligase E3A (*UBE3A*) gene expression in mature neurons of the brain (1, 2). The paternal copy of *UBE3A* is expressed in most peripheral organs, potentially leading to haploinsufficiency in these tissues. However, due to brain-specific imprinting, paternal *UBE3A* is silenced in the central nervous system (CNS) by a long noncoding antisense transcript (*UBE3A-ATS*), resulting in a complete loss of UBE3A expression in the brain (3). This genetic configuration in AS leads to microcephaly, severe developmental delays, deficiencies in expressive communication, a typical facial appearance, deficits in movement and coordination, hypotonia, generalized epilepsy, sleep disturbances, and other characteristic behaviors, such as frequent smiling and laughter (4). In addition to the effects on neurodevelopment, many caregivers report gastrointestinal (GI) issues in AS patients. Particularly, children with AS are often reported as poor feeders due to hypotonia of the throat (5), and they have a high rate of constipation (6). Despite this strong association of GI disorders in AS patients, the mechanisms underlying this remain largely unknown.

The microbiota-gut-brain axis represents the bidirectional communication pathways that connect the gut-microbiota to the brain and modulate behavior (7). Studies using germfree (GF) mice have identified multiple behavioral impairments, including cognitive deficits (8) and anxiolytic behaviors (9), compared to colonized controls, supporting a role for gut microbes in maintaining these behaviors. Regulation of behaviors by gut bacteria might occur via a combination of multiple pathways, including endocrine signaling through hormones and neuro-active metabolites, as well as signaling via the immune system and vagus nerve. Colonization of the gut microbiome begins at birth and plays a critical role in building a healthy gut, shaping immune processes, and neurodevelopment (10, 11).

The disruption of microbial communities in the GI tract has been implicated in a number of different neurodevelopmental and neurodegenerative disorders, such as autism spectrum disorders (12, 13), Alzheimer's disease (14, 15), Parkinson's disease (16–18), depression (19), amyotrophic lateral sclerosis (20–22), schizophrenia (23, 24), and attention deficit hyperactivity disorder (25). Those with monogenetic neurodevelopmental disorders may also exhibit changes in microbial composition that may explain some of the GI symptoms seen in subsets of patients. For example, Rett syndrome, a severe and progressive X-linked neurological disorder affecting mainly females due to mutations in the *MECP2* gene, has a strong association with GI dysfunction, including intestinal dysbiosis characterized as a disruption to the bacterial homeostasis (26–28). Relevant to the work herein, the same region on chromosome 15 that is affected in AS causes Prader-Willi syndrome (PWS) when the paternal contribution of genes on chromosome 15 is lost. Although clinically distinct from AS, individuals with PWS exhibit obesity, hyperphagia, and reduced metabolic rate, in the context of an altered microbiome (29–31). The frequency and scope of GI illnesses in AS, however, have never been studied and the diagnostic consensus estimates that the prevalence may affect up to 70% of individuals with AS (6). GI problems in AS were reviewed using medical records of 163 individuals with AS with different genetic subtypes and characterized, identifying at least one GI dysfunction in most patients (6). The two most common dysfunctions were constipation and gastroesophageal reflux disease (GERD). Other GI problems reported included cyclic vomiting episodes, difficulty swallowing, excessive swallowing, and eosinophilic esophagitis (6). Despite this prevalence of GI symptoms in AS patients, a 16S rRNA sequencing study in AS patients has not yet been performed.

To study the potential impacts of AS on the brain and the gut microbiota, three AS animal models that lack maternal UBE3A expression, like humans, were compared. The mouse model contains an inserted nonsense mutation in exon 2 of the mouse *Ube3a* gene (32), whereas the rat and pig models have a full gene deletion from the use of CRISPR/Cas9 nucleases flanking the *Ube3a* gene (33–35). We compared the global gut microbial community (alpha and beta diversity) as well as the microbial composition in all three animal models. In addition, to better understand the underlying metabolic processes affected by changes in the microenvironment of the gut microbiota in genetic animal models of AS, an inference analysis of metabolic pathways based on the bacterial microbiome was performed. While similar changes were seen at the phylum level in AS animals compared to controls, distinct patterns were observed in each species.

## RESULTS

### The global microbial community structure in AS animal models is specific to each species.

**FIG 1:**
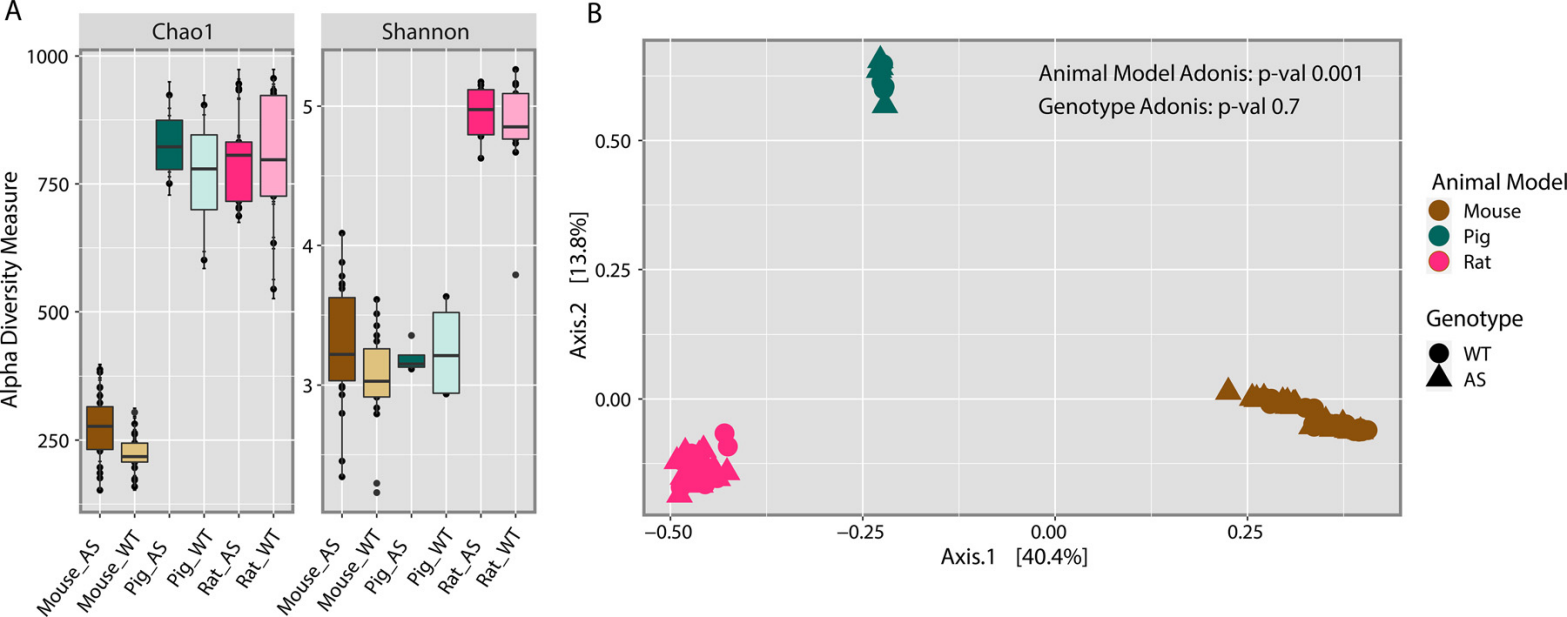
Microbial community structures differed between the animal models. (A) Alpha diversity analysis for both richness (Chao1) and diversity (Shannon) indexes and Kruskal-Wallis pairwise comparison were performed to determine the differences between the overall animal models. Rat microbial communities were significantly richer (*P = *6.992939e−12) and more diverse (*P* = l 8.370003e−12) than those in mice. Rat communities were also more diverse than pig communities (*P = *2.417261e−05), while pig microbial communities were richer than those in mice (*P = *9.155458e−06). (B) Beta diversity analysis using the Bray-Curtis dissimilarities index showed significant dispersion of the samples by animal model (Adonis *P = *0.001).

Richness and diversity measures across all species were performed. These findings indicated that the mouse model is much less rich in total bacterial numbers than either pig or rat models (Fig. 1A). In contrast, the diversity index showed that both mouse and pig models were similar in diversity, whereas the rat model was significantly higher in diversity (Fig. 1A). Significant dispersion based on animal model (*P* < 0.001) was observed; however, there was no significant separation based on genotype (*P* = 0.7) (Fig. 1B).

**FIG 2:**
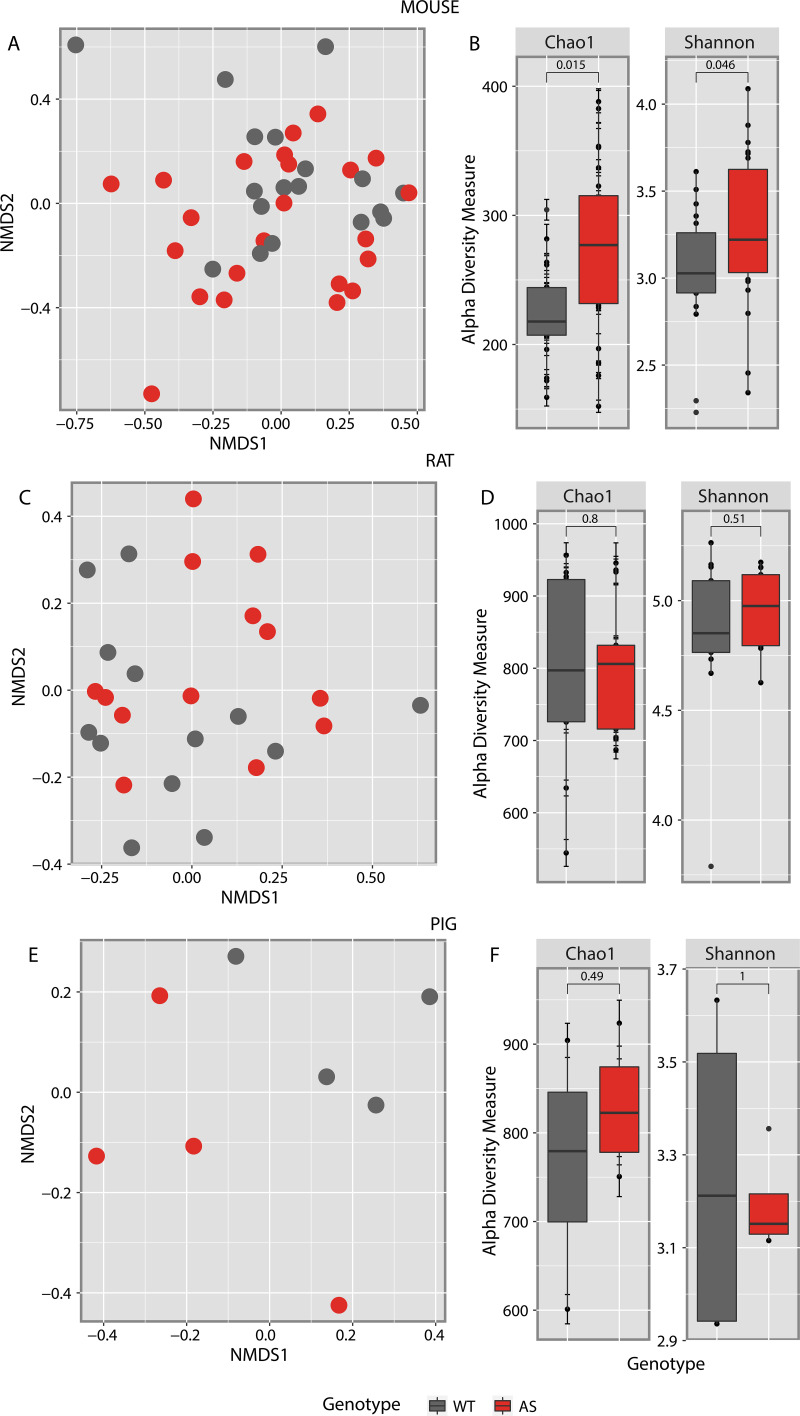
Individual animal models' overall gut microbial composition structures were not altered by AS genotype. The Bray-Curtis dissimilarities index was used to analyze the dispersion of samples using an NMDS visualization. (A and B) The mouse model presented no significant dispersion of the samples (A), but alpha diversity showed the compositions in AS mice were richer (*P = *0.015) and more diverse (*P = *0.046) than in WT mice (B). (C to F) Rat and pig models showed no significant dispersion (C and E), and there were no significant differences in alpha richness or diversity indexes (D and F).

Individual animal model analyses were performed to assess specific differences in the microbial community structures between healthy (wild-type [WT]) and AS animals for each species. The microbial community structure of individual animals relative to the genotype identified no significant differences between genotypes across all three species (Fig. 2A, C, and E). Alpha diversity analysis exploring the richness and diversity index of the individual species identified significant differences in mice, with both Chao1 and Shannon indexes supporting a richer (*P* < 0.01) and more diverse (*P* < 0.05) microbiome in AS mice compared to WT controls (Fig. 2B). In contrast, no significant differences were identified in either pig or rat between AS and WT animals (Fig. 2D and F).

### Overall microbial composition differences across multiple AS animal models.

**FIG 3:**
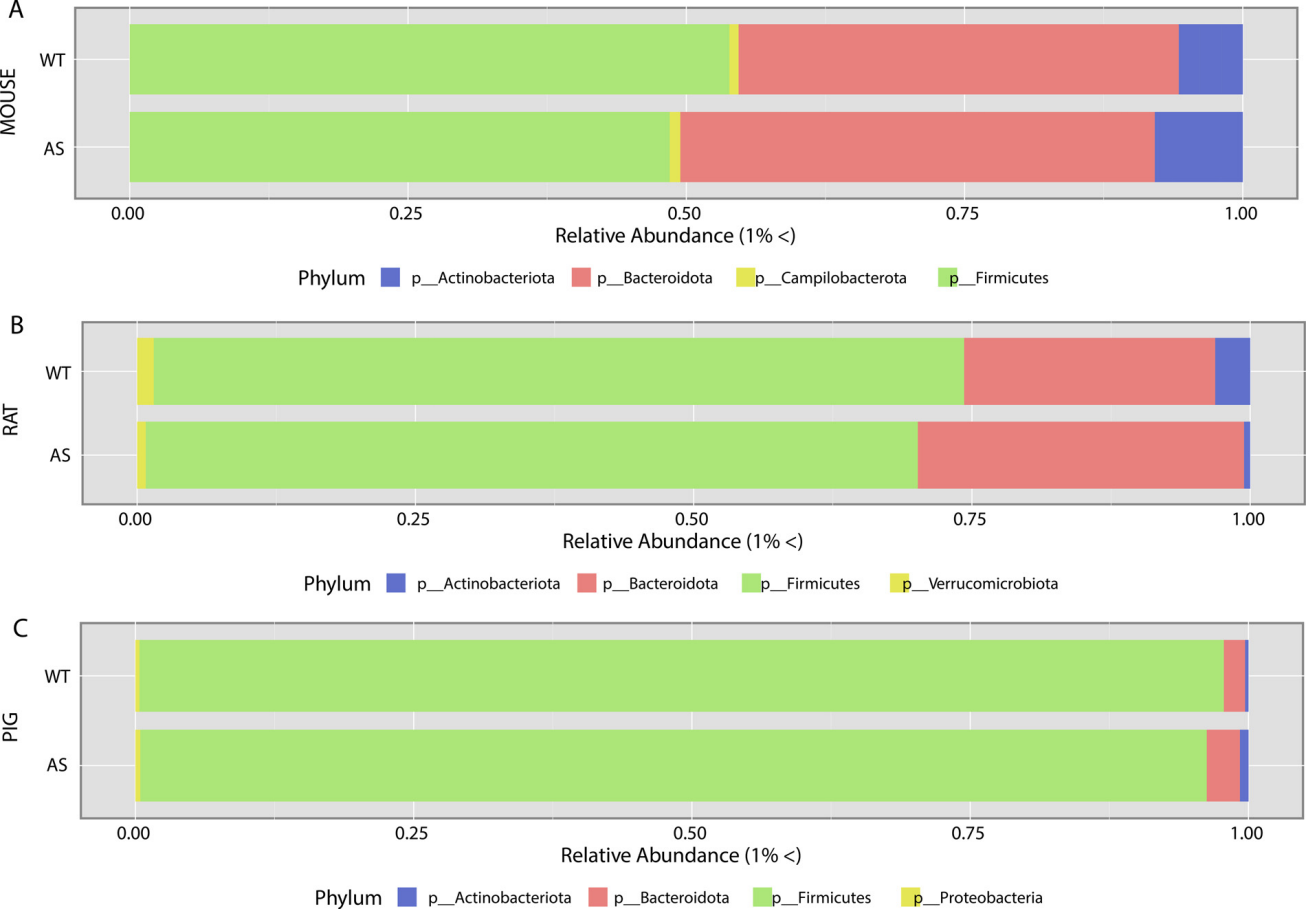
Phylum-level differences between AS and WT microbial compositions by animal model. All panels represent phyla composition present above 1% relative abundance. A comparison of overall phyla composition between the animal models (mouse [A], rat [B], and pig [C]) and genotype (AS and WT) showed a similar decrease of *Firmicutes* and an increase of *Bacteroides* across all species.

To characterize the differences between the most abundant operational taxonomic units (OTUs) in each genotype, the relative abundances of all OTUs were calculated, and only phyla and genera with a minimum of 1% relative abundance were included in the analysis. Phylum-level differences in relative abundance were consistent between the AS and WT groups in all animal models (Fig. 3). Across the three AS animal models, a reduction of *Firmicutes* (green) and an increase of *Bacteroidota* (red) were observed compared to WT controls; these represent the major phyla for all three animal models (Fig. 3A to C). In contrast, an increase of *Actinobacteriota* was identified in both AS mice and pigs, which was reduced in the AS rats in comparison to WT (Fig. 3B). These differences at the phylum level suggested that the AS genotype disrupts the abundance of the most highly abundant phyla in the gut, with similar changes observed across multiple species.

**FIG 4:**
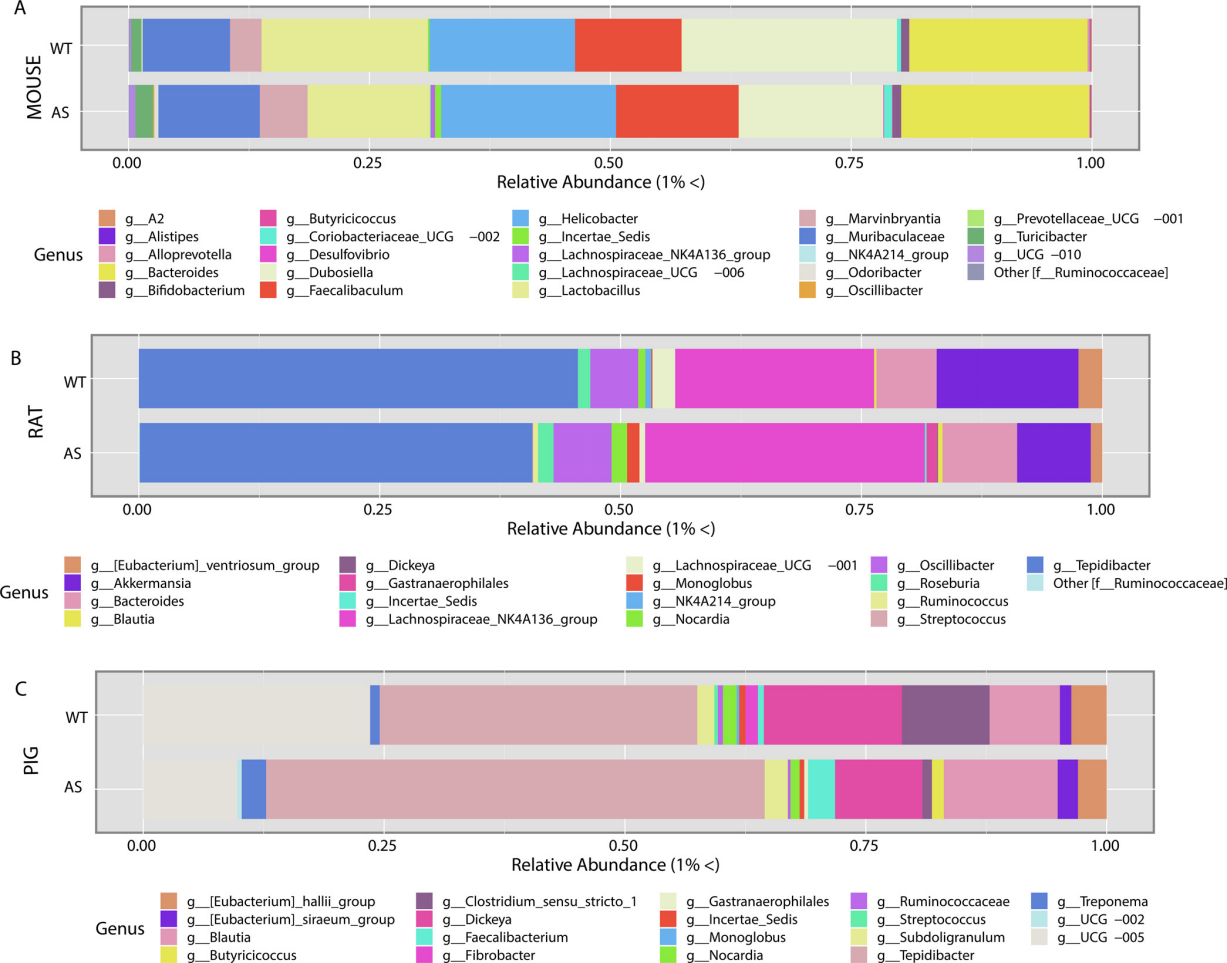
Genus-level differences between AS and WT microbial compositions by animal model. Relative abundance of genera above 1% showed different genus compositions across all animal models. (A) Mice showed a decrease in *Dubosiella* and *Lactobacillus* and an increase in *Helicobacter* and *Bacteroides* for AS versus WT animals. (B) In rats, we saw an increase in the *Lachnospiraceae* NK4A136 group and a decrease in *Akkermansias* in AS versus WT animals. (C) Finally, in the pig model, we saw an increase in *Tepidibacter* and *Blautia* and a decrease in *Clostridium sensu stricto 1* and *Lachnospiraceae* UCG-005 in AS versus WT animals.

At the genus level, all AS animals demonstrated differences in abundance compared to their respective WT control. For example, in AS mice, increases of *Bacteroides*, *Coriobacteriaceae* UCG-002, *Faecalibaculum*, *Helicobacter*, Incertae sedis, *Lachnospiraceae* NK4A136 group and UCG-006, *Marvinbryantia*, and *Turicibacter* were observed relative to WT control mice (Fig. 4A). These increases were accompanied by reductions of *Lactobacillus* and *Dubosiella* (Fig. 4A). In AS rats, an increase in *Bacteroides*, *Blautia*, *Gastranaerophilales*, *Monoglobus*, *Nocardia*, *Roseburia*, and *Ruminococcus* was seen (Fig. 4B). This was coupled with a decrease in *Akkermansia*, Eubacterium ventriosum group, *Tepidibacter*, and *Lachnospiraceae* UCG-001 compared to WT controls (Fig. 4B). Finally, AS pigs had increased *Subdoligranulum*, *Tepidibacter*, Treponema, *Faecalibacterium*, *Blautia*, and *Butyricicoccus*, whereas there was a decrease in UCG-005, *Clostridium sensu stricto1*, *Fibrobacter*, *Monoglobus*, and Streptococcus compared to WT control pigs. While species-level characterization is preferable to establish the specific role of each organism in the gut, such genus characterizations can provide an important picture of how a genetic impairment affects the microbial composition (36). Taken together, the analyses of all the animal models showed differences between the AS and WT animals at the genus level, but these differences did not overlap across models, in contrast to the findings at the phylum level, highlighting the differences in composition across each species.

### Possible bacterial biomarkers identified across and within AS models.

**FIG 5:**
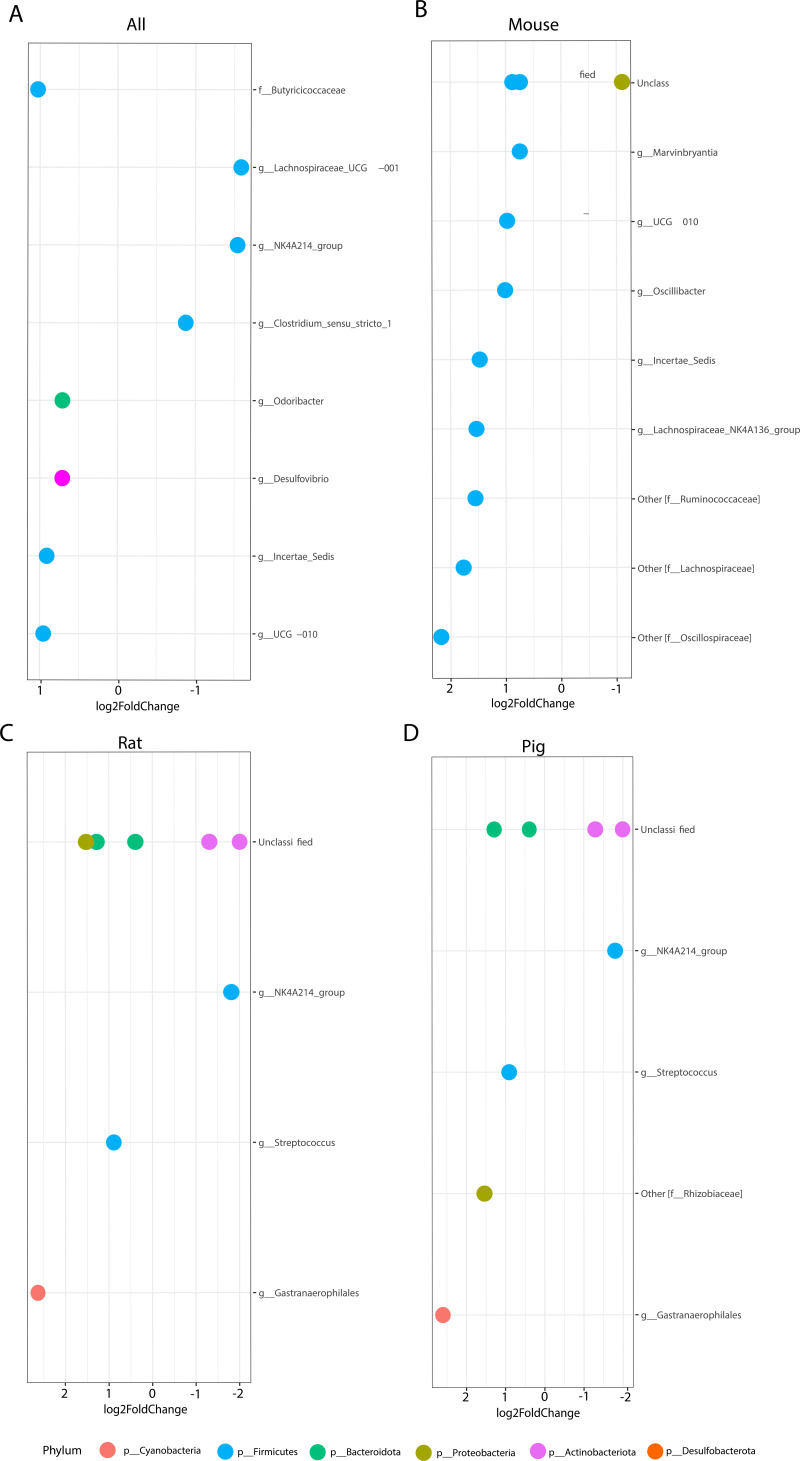
Fold change analysis showed significantly different bacterial groups associated with AS. This analysis only considered bacteria that showed significant differences between genotypes (*P < *0.05). (A) Overall differences between genotypes across the microbiome of all animal models. Values above 0 correlated with AS, and values below 0 correlated with WT. The bacterial groups associated with AS in individual animal models (mouse [B], rat [C], and pig [D]) are shown.

Due to the wide differences in genus-level taxonomic compositions found between the different animal models, a fold change (Deseq2) analysis was performed based on genotype and separated by animal model. This analysis helped establish specific high- and low-abundance taxa that were differentially abundant within the microbial ecosystem according to genotype. Analysis of AS versus WT across all three animal models identified a differential abundance of *Desulfobacterota*, *Bacteroidota*, and *Firmicutes* genera based on genotype. Genus-level differences showed a higher prevalence of UCG-010, Incertae sedis, *Desulfovibrio*, *Odoribacter*, and *Butyricicocaceae* family members in AS animals (Fig. 5A). In contrast, WT animals had differential abundances of *Clostridium sensu stricto*, NK4214 group, and *Lachnospiraceae* UCG-001 (Fig. 5A).

While these differences accounted for the genotype in all the animal models, there were also innate differences between the gut microbiome of all three species. Therefore, fold change analysis was performed in all the models individually by genotype. In mice, a differential abundance of Incertae sedis, *Oscillibacter*, UCG-010, *Marvinbryantia*, *Lachnospiraceae* NK4A136 group, and other unclassified genera pertaining to *Oscillospiraceae*, *Laachnospiraceae*, and *Ruminococaceae* families were identified in the AS model compared to WT controls (Fig. 5B). In the rat and pig models, fewer bacterial genera were differentially abundant in AS animals, these being *Gastranaerophilales*, Streptococcus, and *Rhizobiaceae* families (only in pigs) and some unclassified *Bacteroidota* and *Proteobacteria* (only in rats) in the phylum-level comparison to WT controls (Fig. 5C and D). WT rats and pigs presented with NK4A214 group and unclassified *Actinobacteria* both highly prevalent compared to AS animals (Fig. 5C and D). These findings suggested similarities between pig and rat microbial ecosystems and illustrated how the AS genotype affects the intestinal bacterial community.

### Differential bacterial metabolic pathways identified in each animal model of AS.

**FIG 6:**
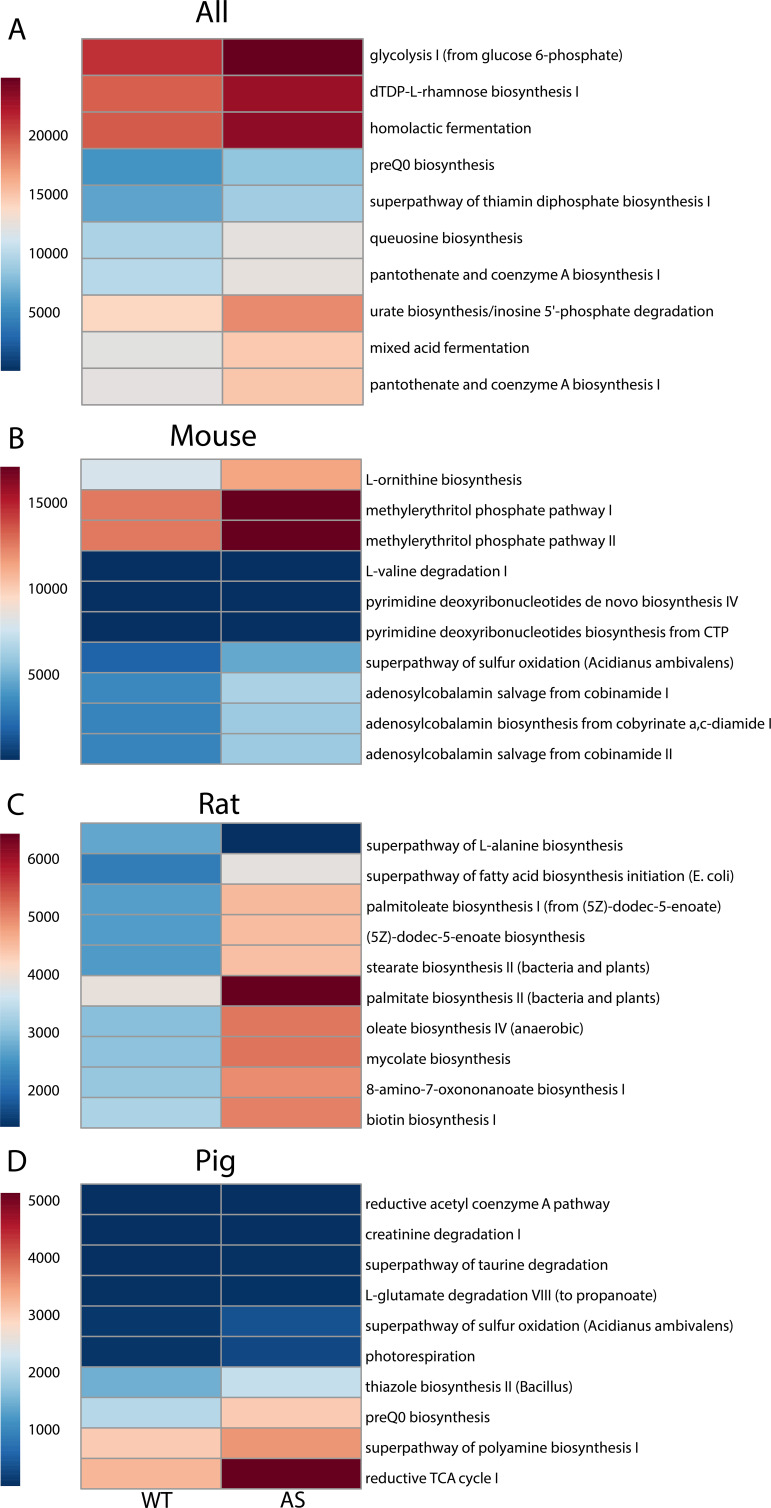
Metabolic pathway prediction analysis showed significant differences between genotype and animal model. Prediction of metabolic pathways based on the microbial ecosystem was analyzed, and pathways significantly different overall (A) or in individual animal models (mouse [B], rat [C], pig [D]) showed no overlap, but similar functional correlations were related to vitamin synthesis and utilization.

To better understand the underlying metabolic processes affected by changes in the microenvironment of the gut microbiota in genetic animal models of AS, an inference analysis of metabolic pathways based on the bacterial microbiome was performed using PICRUST. The metabolic pathway analysis was performed for both individual and combined species, which allowed visualization of important pathways that play a role in each separate animal model and pathways that are impacted specifically due to genotype. The combined model analysis suggested that the microbiota in AS animals has higher activity in metabolic processes such as glycolysis, lactic fermentation, glycan building blocks, nucleoside biosynthesis, vitamin B_1_ synthesis, vitamin B_5_, and coenzyme A biosynthesis and urate production and accumulation (Fig. 6A) compared to WT controls. These results suggested changes in the metabolic pathway activity occur based on genotype, yet preexisting differences based on each animal model can introduce variability.

In the mouse, metabolic pathway analysis reflected possible changes based on genotype. Evidence of increased activity of amino acid biosynthesis, isopentenyl diphosphate synthesis, adenosylcobalamin salvage, and biosynthesis from vitamin B_12_ analogs (cobinamides) was seen in AS mice compared to WT controls (Fig. 6B). In the rat, similar increases in the biosynthesis of amino acids, lipokine biosynthesis (palmitoleate), fatty acid synthesis, and biotin biosynthesis were observed in AS rats (Fig. 6C). Finally, in the pig model, evidence for increased biosynthesis of thiazole (vitamin B_1_), production of polyamines, and changes in the reductive tricarboxylic acid (TCA) cycle (responsible for making organic molecules to produce sugars, lipids, amino acids, etc.) in AS animals were seen compared to WT controls (Fig. 6D).

Overall, when the individual metabolic differences across the three animal models were compared, possible similarities among amino acid biosynthesis pathways were observed across species. Additionally, all models showed evidence of increased activity in different pathways related to the vitamin B complex and its involvement in biological processes, suggesting a common feature of AS across animal models.

## DISCUSSION

In the past decade, there has been a significant increase in research on AS, from basic to applied research, due in part to a large effort to create preclinical animal models (37) for the purpose of identifying targeted treatments and outcome measures for future clinical trials. Given that GI symptoms are commonly seen in patients and can significantly impair their quality of life, the identification of factors that can regulate GI physiology is critical in advancing these goals. The gut microbiome is crucial for the establishment of GI physiology and function, and alterations in the colonization of the gut microbiome are prevalent in neurodevelopmental disorders. Characterizing the impacts of *Ube3a* deletion on the gut microbiome using animal models has the powerful advantage to control for variables that are challenging in humans, including environment and diet. As there are currently no gut microbiome studies reported in AS patients, this study represents the first attempt to characterize the microbiome in AS, using animal models, and to identify pathways that could be targeted to improve GI pathophysiology in patients. Here, we used 16S ribosomal DNA amplicon sequencing in AS animal models in three species to uncover common colonization features associated with maternal *Ube3a* deletion.

In the current study, we first looked at the biodiversity at different scales, both within each model and common across all three models. The mouse model was found to be much less rich and diverse in comparison to the other two AS models. The lack of diversity and richness, as measured by alpha diversity, has been reported before in laboratory mice compared to wild Mus domesticus and is likely in part due to the standard diet which they are provided (38). However, based on beta diversity, the mouse microbiota seems to be closer to that of humans than that of rats (39). No major differences in the microbial structure were observed in AS animal models compared to WT controls, which suggested that the overall primary microbial community remained conserved. Given that only bacteria with a >1% overall abundance were assessed, it is possible that the changes could be occurring in low-abundance bacterial groups that were not captured in this analysis. Alterations in less-abundant bacteria can potentially change interactions within the gut without affecting the overall microbial community.

Findings from the highly abundant taxonomic groups suggested that the main differences between the AS animals and WT controls resided in a reduction of lactic acid bacteria, which are essential in the gut for maintaining health (40–43). The decrease of *Bifidobacterium* coupled with an increase in the abundance of *Bacteroides* was seen in AS animals, and this has been previously observed in patients with chronic constipation. As typically seen in constipation studies, there was no direct consensus on whether these changes in the gut microbiome are causal or the result of a side effect of the condition. However, constipation is the most common symptom seen in AS patients, and these findings support a change in the microbial composition associated with the AS genotype. Observing these trends in taxonomic groups within three different animal models of AS further supported that these differences arise due to genetic deletions resulting in AS.

Similar to AS, PWS is a neurodevelopmental disorder associated with intellectual disability and behavioral problems and it affects the same region on chromosome 15q11-q13. PWS patients also have a high prevalence of constipation (44, 45) and often display hyperphagia and obesity. While several recent microbiome studies have shown that while PWS children display a similar alpha diversity and compositions of microbiota compared to healthy children, 13 bacterial genera have been identified to be differentially abundant (31). Adult PWS patients exhibited an even more distinctive microbiota profile, with low *Blautia* and enhanced RF39 (phylum *Tenericutes*), *Ruminococcaceae*, *Alistipes*, *Erysipelotrichacaea*, *Parabacteriodes*, and *Odoribacter* (29). The microbiota has been the target in at least two clinical trials (NCT03277157 and NCT03548480) for PWS; in these studies, the daily consumption of probiotics such as Bifidobacterium animalis subsp. *lactis* B94 (45) and Bifidobacterium animalis subsp. *lactis* strain BPL1 (46) was analyzed for effects on stool frequency, stool form, and other GI symptom improvements. For the BPL1 treatment, it was reported that there were reductions in abdominal adiposity in a subgroup of PWS individuals that were older than 4.5 years, and BPL1 improved fasting insulin concentration and insulin sensitivity (46). Furthermore, those authors observed modest improvements in some mental health symptoms. Finally, another syndrome arising from the same genomic region as and PWS is Dup15q syndrome. The duplication or extra copy of the region 15q11. 2-q13 seems to be less commonly reported but is also associated with GI problems. Despite the low prevalence and sparse literature, based on a recent study it is apparent that studying the relationship between GI symptoms, sleep problems, comorbid psychopathology, ASD symptoms, and challenging behavior in Dup15q patients and how these conditions can shape the Dup15q phenotype is of high importance for future studies (47).

The GI tract communicates bidirectionally with the brain and is closely associated with neurodevelopment, as both develop during early neonatal life in multiple animals. Altered colonization of the gut microbiota, termed “dysbiosis,” has been observed to correlate with autism spectrum disorder (ASD) (13). In ASD, behavioral and neurodevelopmental changes have been correlated with a reduction of *Bifidobacterium* and *Blautia* species (48), similar to the changes in the AS animal models seen here, compared to WT controls. These findings suggest that these microbial community impairments may serve as the main contributors to neurodevelopmental delays. The abundance of other bacterial species, including *Desulfovibrio*, *Lactobacillus*, and *Bacteroides*, are also increased in ASD (48, 49). Our fold change analysis presented an overall increased prevalence of *Lachnospiraceae* Insertae sedis, *Desulfovibrio*, and *Odoribacter* in AS in comparison to WT controls. Increased *Lachnospiraceae* Insertae sedis has been associated with multiple diseases, including major depressive disorder and nonalcoholic fatty liver disease (50). Moreover, *Desufovibrio* has been correlated with Parkinson’s disease, and its abundance in the gut is directly correlated with disease severity (51). *Odoribacter* has been correlated with attention deficit/hyperactivity disorder and destabilizes the levels of dopamine and serotonin in the gut (52). This suggests that the observed bacterial groups increased in AS align with findings from current studies of other neurodevelopmental diseases (53). While these overall trends in our preclinical data resemble the current literature in similar neurodevelopmental syndromes in humans, variability of abundances within individual samples across groups, due to age or caging effects, could skew the data. While these results provide helpful information as to what organisms might be affected in AS patients, the lack of consensus across all samples and animal models prevents the identification of unique bacteria as biomarkers of health or disease for AS.

Prediction of the metabolic function changes resulting from the altered gut microbiota in AS animals allowed us to understand the metabolic implication of dysbiosis in AS. As explored in the inferred metabolic pathways analysis using PICRUSt2, changes in vitamin B_12_ synthesis and utilization are impacted due to microbial dysbiosis. Since vitamin B_12_ has the potential to break down homocysteine, increased B_12_ will increase homocysteine, which is directly correlated with dementia, heart disease, and stroke (54). In mice, deficiency of B_12_ is associated with protection against colitis (55), while in rats, B_12_ deficiency causes intestinal barrier defects (56). Cobalamin or B_12_ deficiency can cause increased homocysteine, or hyperhomocysteinemia, which occurs commonly in patients with inflammatory bowel disease (57). Similar changes in the gut microbial ecosystem in ASD studies have revealed the implications of gut dysbiosis in the production and utilization of vitamins such as B_12_ (58). The use of comparisons between AS and ASD that lead to microbial dysbiosis and metabolic disparities has the potential to identify what changes in the metabolome and microbiome contribute to disease severity.

In conclusion, the microbial composition analysis of AS within three separate animal models showed prominent changes in the composition and metabolic capacity of the gut microbiome compared to those in WT control animals. Bacterial groups that were significantly altered within the AS models have also been correlated with other neurodegenerative and GI diseases, highlighting their important role in gut-brain communication. It remains to be determined whether changes to the gut microbiome are a cause or effect of the GI AS symptoms, but the current analysis suggests that the microbial ecosystem may promote adverse gut-brain pathways. Future studies assessing impacts of altered microbial composition on GI physiology and motility in these animal models of AS are highly warranted based on these current findings. Future longitudinal studies are also warranted to assess both early colonization patterns resulting from the maternal microbiota and associations between phenotype and disease across the life span in these animal models. Beneficially modulating the gut microbiome may serve to improve both neural and gastric symptomatology in patients with AS, possibly improving overall quality of life.

## MATERIALS AND METHODS

### Animals.

Food and water were provided *ad libidum* for all animals. In total, 41 mice, 26 rats, and 8 pig fecal samples from both sexes were collected for this study (*N* = 75). Mice were 13 to 25 weeks of age, rats were 8 weeks of age, and the pigs were 17 weeks old at the time of the fecal sample collection. All animals were housed in appropriate light-dark conditions and fed standard food and water according to the model’s dietary needs. Mice and rats received Teklad Global 18% protein rodent diet 2918 (Envigo, Hayward, CA, USA). Pigs received MG Pig Starter 20% Gen 2.0 for pigs weighing up to 44 pounds, and adults received MG Hog Pellets (M-G, Inc., Feed Division, Weimar, TX, USA).

**(i) Mice.** Mice used in the study were B6.129S7-*Ube3a^tm1Alb^*/J (Jackson Laboratory; strain 016590) (32). WT C57BL6/J littermates served as controls. Mice were housed as littermates in a temperature-controlled vivarium maintained on a 12:12-h light-dark cycle in Techniplast cages (West Chester, PA) with Teklad 1/8 corncob bedding (Envigo, Indianapolis, IN).

**(ii) Rats.** We used rats from the recently characterized *Ube3a*^m^*^/p+^AS rat model, which have a full 90-kb deletion of the maternal *Ube3a* gene on a Sprague-Dawley background (33, 34), and WT littermates served as controls. Rats were housed as littermates on a 12:12-h light-dark cycle in Super Rat 1400 ventilated racks and cages (Lab Products Inc., Seaford, DE, USA) with Teklad bedding (Envigo).

**(iii) Pigs.** The AS pig model (Sus scrofa) contained a full 97-kb deletion of the maternal *UBE3A* gene on a mixed Yorkshire/Landrace background (S. V. Dindot, personal communication). Pigs were housed according to genotype in isolation buildings, which were 3-m by 4.5-m solid smooth flooring rooms with rubber stall mats on a 14:10-h light-dark cycle.

### Ethics statement.

All procedures were approved by the Institutional Animal Care and Use Committee of the University of California, Davis (protocols 21644 and 21814) or Texas A&M (protocol 2020-0213) and conducted in accordance with the National Research Council’s *Guide for the Care and Use of Laboratory Animals* (59).

### Fecal sample collection.

Fecal samples from mice and rats were collected at the University of California, Davis. To collect the fecal samples, animals were placed individually in an empty sterile cage for 5 min, and freshly dropped fecal pellets were collected aseptically into sterile tubes using sterile pipette tips or sterile tweezers. Samples from pigs were collected at Texas A&M University. The samples were collected after euthanasia using a sterile fecal loop, placed in sterile tubes, and stored at −80°C until further processing.

### 16S Illumina sequencing.

DNA was extracted from 20 to 40 mg fecal matter using the QIAamp Powerfecal kit (Qiagen). The library preparation and sequencing were performed by the Host Microbe Systems Biology Core at UC Davis using primer pair 341F and 806R in a 300-bp paired-end run for the V3-V4 hypervariable regions of the 16S rRNA gene on an MiSeq system (Illumina, San Diego). The 16S rRNA gene sequencing raw FASTQ sequence files were deposited and processed in QIITA (60) using per-sample FASTQs with a Phred offset of 33, min_per_read_length_fraction of 0.75, and default parameters for error detection using Split libraries FASTQ. Sequences were trimmed to 250 bp, and possible errors of sequencing were filtered using DEBLUR with default parameters. This filtering method substantially reduces computational demand by removing false positives, with stable detection, and it is limited only by library size and sequence diversity (61). Reference OTUs were defined using the SILVA reference database (version 138-99-515-806) with a minimum similarity threshold of 97% and corresponding taxonomy assignment using the default parameters in QIITA. Singletons (OTUs with less than three reads), sequences matching chloroplasts or mitochondria, and unassigned sequences were removed from downstream analyses, followed by a rarefaction to the minimum library size, which was 21,907 (62). The main variable utilized for analysis was genotype, WT control or AS, with each species assessed individually or compared to each other in single-group analysis. Considering the limitations and poor resolution of species-level characterizations with 16S rRNA genomic sequencing (63), the analysis presented here mainly focused on phylum- and genus-level differences.

Alpha diversity, beta diversity, and taxonomic composition plots were built using R v. 4.2, ggplot2 v. 3.3.6, and phyloseq v. 1.38.0 (64, 65). Beta diversity analyses of microbial communities were performed by computing the pairwise Bray-Curtis distances (66) between samples and plotted using nonmetric multidimensional scaling (NMDS). To determine the significance of the dispersion between the samples, the results of analysis dissimilarities were calculated directly from the distance matrix with ADONIS. Alpha richness (Chao1, estimated number of OTUs) and diversity (Shannon, equitability) (67) indexes were used to establish significant differences between the genotypes and animal models, which were assessed with the nonparametric Wilcox test. Significance was defined as a *P* level of <0.05. To establish significant differences between specific OTUs, we applied a fold change analysis using the Deseq2 pipeline (68) to visualize possible bacterial biomarkers related to the genotype in all the samples, in the separate models. While fold change analysis can elucidate trends within a microbiome in a given group, overall abundance of a given identified taxon might be skewed by individual samples with high abundance and not comparable across the entire sample group. Therefore, these results served to generate hypotheses and aid in understanding key bacterial groups that remain poorly described and can be targeted in future studies of AS.

Abundance was characterized at the phylum and genus levels across the three animal models. A stringent method of filtering was applied using DEBLUR, which substantially reduces computational demand, removes false positives, provides stable detection, and is only limited by library size and sequence diversity (61). Phylum-level abundance was chosen for characterization, allowing determination of broad changes in the microbial ecosystem, while genus-level abundance was selected to aid in understanding more specifically the role these genera might be play in the ecosystem (69). Finally, 16S rRNA sequencing is known to be reliable for both family and genus classification, with poor resolution at the species level (63).

### PICRUSt metabolic analysis.

Metabolic pathway inference analyses were performed using PICRUSt2 software (70). A pathway-level inference analysis was performed, in which MetaCY pathways were inferred using enzyme classification number of abundances. Output used for analysis was composed of an unstratified (sum of all sequences contributing to OTUs) pathway abundance table. Analysis was performed using Metaboanalyst software (71), and pathways were filtered by the mean intensity and log transformation of the abundance counts. Subsequently, the ward clustering method was used, and Euclidean distances to group heatmaps are presented only for significantly different pathways identified. Significance was calculated using a *t* test or analysis of variance as appropriate. While results obtained using PICRUSt2 can identify trends of possible functional pathways that differ across AS models, it represents an analysis bias as it is based on available reference genomes, which limits the identification of less abundant or rare pathways that might be of interest. Moreover, it also cannot provide strain-specific functionality due to the level of characterization that is reached (70). For this matter, while highly interesting, these results require further testing and will serve in developing new hypotheses to this understudied model in future studies.

### Data availability.

The data generated in this study are publicly available in Qiita under the study ID 14071. Sequence data associated with this study can be found under EBI accession ERP139429.
